# Relevance of charged and polar amino acids for functionality of membrane toxin TisB

**DOI:** 10.1038/s41598-024-73879-7

**Published:** 2024-10-03

**Authors:** Florian H. Leinberger, Bork A. Berghoff

**Affiliations:** 1 Institute for Microbiology and Molecular Biology, Justus Liebig University Giessen, 35392 Giessen, Germany; 2 Present Address: Institute of Molecular Biology and Biotechnology of Prokaryotes, University of Ulm, 89069 Ulm, Germany

**Keywords:** Type I toxin-antitoxin systems, Pore formation, Membrane depolarization, ATP depletion, Antibiotic persistence, Microbiology, Molecular biology, Physiology

## Abstract

Bacterial dormancy is marked by reduced cellular activity and the suspension of growth. It represents a valuable strategy to survive stressful conditions, as exemplified by the long-term tolerance towards antibiotics that is attributable to a fraction of dormant cells, so-called persisters. Here, we investigate the membrane toxin TisB (29 amino acids) from the chromosomal toxin-antitoxin system *tisB/istR-1* in *Escherichia coli*. TisB depolarizes the inner membrane in response to DNA damage, which eventually promotes a stress-tolerant state of dormancy within a small fraction of the population. Using a plasmid-based system for moderate *tisB* expression and single amino acid substitutions, we dissect the importance of charged and polar amino acids. We observe that the central amino acids lysine 12 and glutamine 19 are of major importance for TisB functionality, which is further validated for lysine 12 in the native context upon treatment with the DNA-damaging antibiotic ciprofloxacin. Finally, we apply a library-based approach to test additional TisB variants in higher throughput, revealing that at least one positive charge at the C-terminus (either lysine 26 or 29) is mandatory for TisB-mediated dormancy. Our study provides insights into the molecular basis for TisB functionality and extends our understanding of bacterial membrane toxins.

**Supplementary Information:**

The online version contains supplementary material available at 10.1038/s41598-024-73879-7.

## Introduction

Toxin-antitoxin (TA) systems are widely distributed among prokaryotes. They are classified into different types according to the nature of the antitoxin (protein or RNA) and the mode of toxin inhibition. Under non-stress conditions, activity or synthesis of the toxin is prevented by the antitoxin. However, certain events may release inhibition of the toxin, which promotes toxicity by interference with essential cellular functions, resulting in growth inhibition or even cell death^1,2^. TA systems are regularly found on mobile genetic elements (MGEs), such as plasmids and prophages, and contribute to the stable inheritance of these MGEs in expanding populations^1,3^. TA systems are also present in chromosomes, sometimes in fairly high numbers^4^. Even though the biological functions of many chromosomal TA systems are less well understood, it has been observed that they contribute to the defense against bacteriophages or support survival in stressful environments^5,6^.

Type I TA systems are ubiquitous and defined by an RNA antitoxin that translationally represses the toxin messenger RNA (mRNA) by antisense-mediated binding. Under certain stress conditions the toxin mRNA either out-titrates the RNA antitoxin or the RNA antitoxin is depleted. Either way, toxin translation is enabled and toxicity occurs^7^. Type I toxins are usually small hydrophobic proteins with sizes below 50 amino acids (AAs), containing a central transmembrane helix (TMH) as well as short N- and C-terminal extensions. Upon targeting of the inner membrane, they disturb membrane functioning or even modulate cell morphology^7,8^. One well-characterized type I toxin is HokB from the *hokB/sokB* TA system in *Escherichia coli*. HokB has a total size of 49 AAs and a TMH consisting of 23 AAs (positions 7–29). The C-terminal extension is localized in the periplasm and contains a cysteine residue (C46) that is essential for dimerization and pore formation *via* intermolecular disulfide bridges^9^. While intermediate HokB pores are probably narrow and cause breakdown of the proton motive force (PMF) by allowing protons to traverse the inner membrane, mature pores have an effective radius of ~ 0.6 nm and promote ATP efflux^10^, and potentially the movement of several other small molecules and ions. The resulting energy deprivation is expected to induce antibiotic tolerance *via* formation of dormant cells (i.e., persisters)^10,11^.

Another well-characterized type I toxin is TisB from the *tisB/istR-1* TA system in *E. coli*. The *tisB* gene is under LexA control and strongly transcribed under DNA damage (SOS) conditions^12,13^. The RNA antitoxin IstR-1 and structural features within the *tisB* mRNA represent a threshold for TisB production, contributing to phenotypic heterogeneity due to uneven TisB levels among individual cells^14^. TisB has a total size of 29 AAs and a TMH consisting of 20 AAs (positions 6–25). Unlike HokB, the TisB toxin does not contain cysteine residues that might promote dimerization *via* intermolecular disulfide bridges. However, TisB also forms pores and causes PMF dissipation, which is accompanied by ATP depletion and further downstream effects, such as reactive oxygen species (ROS) formation, protein aggregation and cytosolic condensation^15–17^. The concomitant dormancy favors the establishment of a small fraction of antibiotic-tolerant persister cells^14,18^.

TisB contains three positively charged lysines (K12, K26 and K29) and two negatively charged aspartates (D5 and D22), resulting in a net charge of + 1. Different models were suggested to explain the molecular basis for TisB functionality. The ‘anion-selective pore’ model is based on in vitro experiments with planar lipid membranes. The model predicts that TisB forms pores with a relatively small diameter of ~ 0,15 nm and that the net charge of + 1 favors anionic selectivity, likely causing collapse of the PMF because hydroxyl anions migrate from the cytoplasm to the periplasm^19^. The ‘charge-zipper’ model is based on molecular dynamics simulations and predicts that TisB forms an antiparallel dimer-of-dimers that is stabilized by a multitude of salt bridges and hydrogen bonds, involving the two positively charged AAs K12 and K26, the two negatively charged AAs D5 and D22, and the polar AA glutamine Q19^20,21^. The resulting TisB pore is predicted to discharge the PMF *via* direct passage of protons from the periplasm to the cytoplasm. Even though the two models are not necessarily mutually exclusive, they have not been substantiated by analyses in living *E. coli* cells. Here, we apply a plasmid-based system for moderate *tisB* expression and amino acid substitutions to reveal the importance of charged and polar amino acids for TisB functionality. The results are discussed with regard to the two prevailing models.

## Results

### Lysine 12 and glutamine 19 are essential for TisB-mediated cellular effects

**Fig. 1:**
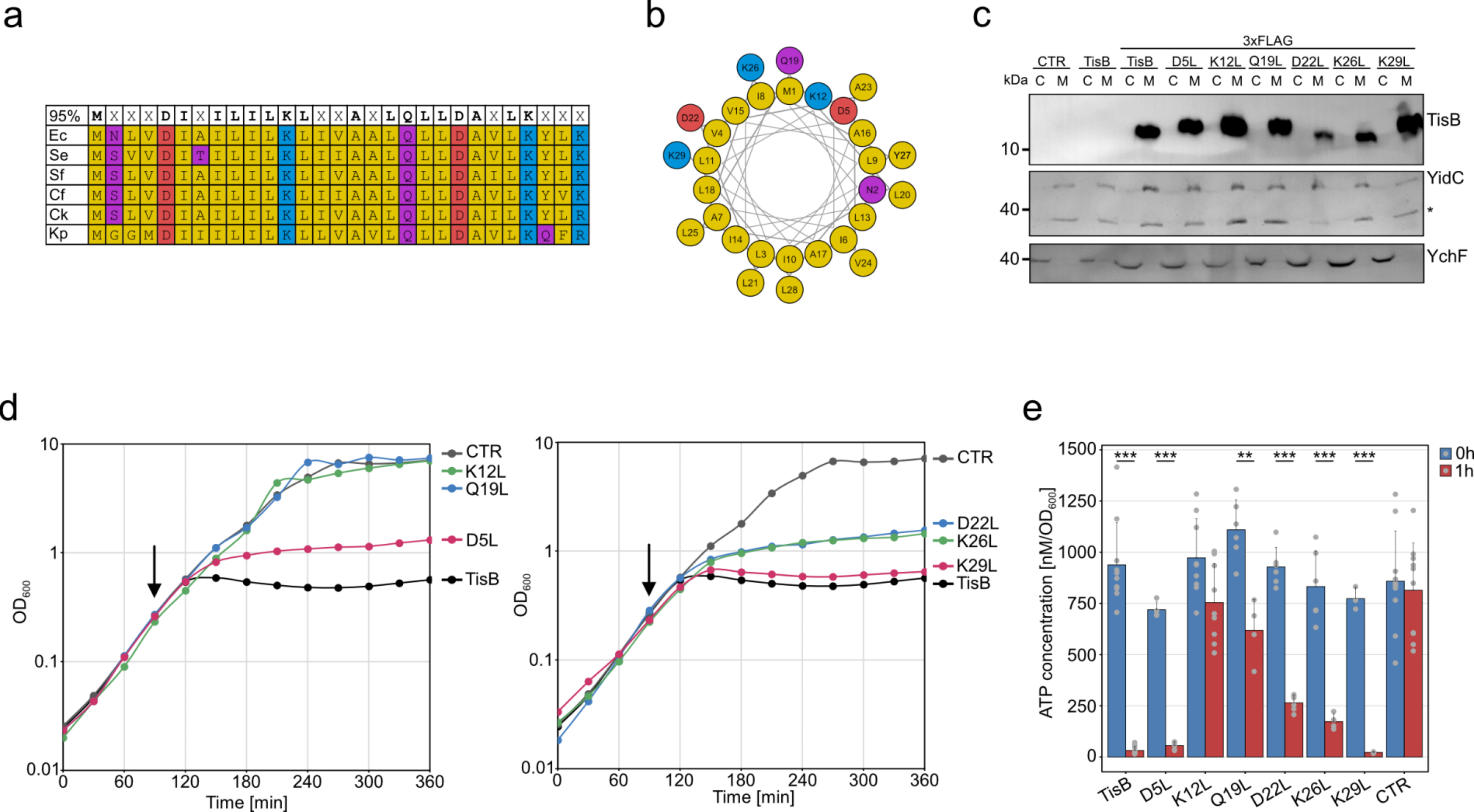
Importance of single amino acids for TisB functionality. (**a**) Conservation analysis of TisB. Conservation levels of TisB were determined *via* BLAST. Amino acids with 95% conservation are in bold. *E. coli* K-12 (Ec), *Salmonella enterica* serovar Typhimurium (Se), *Shigella flexneri* (Sf), *Citrobacter freundii* (Cf), *Citrobacter koseri* (Ck), and *Klebsiella pneumoniae* (Kp). Amino acids color code: nonpolar (yellow), polar (purple), acidic (red), and basic (blue). (**b**) Helical wheel projection of TisB. Same color code as in (a). (**c**) Western blot analysis of TisB localization. Wild type MG1655, harboring p0SD-*3xFLAG-tisB* (3xFLAG-TisB) and variants with different amino acid substitutions, were treated with L-ara (0.2%) during exponential phase for one hour. p0SD-*tisB* (TisB) and an empty pBAD plasmid (CTR) were used as controls. Cytoplasmic (C) and membrane (M) fractions were isolated from total protein samples using ultracentrifugation, followed by Tricine-SDS-PAGE and western blot detection. An anti-3xFLAG antibody was used for detection of 3xFLAG-TisB. Anti-YidC (membrane) and anti-YchF (cytoplasm) antibodies were used as fractionation controls. For unedited western blot images, see Supplementary Fig. S2. (**d**) Growth inhibition by TisB. Wild type MG1655, harboring p0SD-*tisB* (TisB) and variants with different amino acid substitutions, were treated with L-ara (0.2%) during exponential phase (arrow). An empty pBAD plasmid (CTR) was used as control. Data points of OD_600_ measurements represent the mean of at least three biological replicates (TisB: *n* = 9; CTR: *n* = 9; D5L: *n* = 3; K12L: *n* = 3; Q19L: *n* = 3; D22L: *n* = 6; K26L: *n* = 3; K29L: *n* = 3). (**e**) TisB-dependent ATP depletion. Wild type MG1655, harboring p0SD-*tisB* (TisB) and variants with different amino acid substitutions, were treated with L-ara (0.2%) during exponential phase for one hour. An empty pBAD plasmid (CTR) was used as control. Pre- and post-treatment samples were analyzed using a luciferase-based assay to measure cellular ATP levels (nM per OD_600_). Bars represent the mean of at least three biological replicates (TisB: *n* = 7; CTR: *n* = 9; D5L: *n* = 4; K12L: *n* = 9; Q19L: *n* = 4; D22L: *n* = 6; K26L: *n* = 4; K29L: *n* = 3). Error bars indicate the standard deviation. ANOVA with post-hoc Tukey HSD test was performed (*** *p* < 0.001; ** *p* < 0.01).

Toxin TisB is not widely distributed among bacteria and only found within the family of *Enterobacteriaceae* within the class of Gammaproteobacteria^22^. A multiple alignment revealed that the five charged AAs (D5, K12, D22, K26 and K29) and the polar glutamine (Q19) are well conserved among species that are closely related to the *E. coli* wild-type strain MG1655, including *Salmonella enterica*, *Shigella flexneri*, *Citrobacter freundii*, *Citrobacter koseri*, and *Klebsiella pneumoniae* (Fig. 1a). The polar asparagine (N2) was not conserved in the investigated TisB homologs and, therefore, not further considered for investigation. As visualized by a helical wheel projection, the charged AAs and Q19 constitute a hydrophilic face, which is opposed to the hydrophobic face formed by the majority of AAs with a hydrophobic side chain (Fig. 1b). Hence, TisB can be considered as an amphipathic protein. It is intuitive to assume that the hydrophilic faces of several TisB monomers are directed towards each other to form a water-filled pore, whereas the hydrophobic faces are aligned with the lipid bilayer of the inner membrane. Hence, the charged and polar AAs may significantly contribute to TisB functionality, as also suggested by the ‘anion-selective pore’ and ‘charge-zipper’ models^19–21^. To test the significance of individual AAs, we made use of a recently established plasmid-based *tisB* expression system using a Shine-Dalgarno-free upstream region (denoted p0SD-*tisB*), which allows moderate production of TisB by the addition of L-arabinose (L-ara) without causing cell death of *E. coli* MG1655^16^. Even though the expression system does not produce lethal TisB levels, we cannot exclude that it may lead to unwanted side effects due to saturation of the membrane and potential (off-) targets. However, the system is suitable to bypass regulation by the antitoxin IstR-1 and is expected to be sensitive to even subtle changes in TisB activity. The charged and polar AAs were individually substituted with the hydrophobic AA leucine (L) by mutagenesis PCR of the plasmid. Leucine was chosen because it has a molecular weight of ~ 131 Dalton, which is comparable to the substituted amino acids. All TisB variants had a higher grand average of hydropathy (GRAVY) value than native TisB (Supplementary Fig. S1) and were hence expected to localize to the membrane. To confirm membrane localization of the TisB variants, we first constructed the corresponding mutations on plasmid p0SD-*3xFLAG-tisB* for western blot detection of 3xFLAG-TisB in cytoplasmic and membrane fractions. All 3xFLAG-TisB variants were exclusively detected in the membrane fraction, as supported by detection of control proteins YchF (cytoplasmic) and YidC (inner membrane; Fig. 1c). Hence, the AA substitutions had no effect on integration of TisB into the membrane. We noticed that the 3xFLAG-TisB variants differed slightly in their gel migration (Fig. 1c), which was however not reproducible and varied between individual western blot replicates (Supplementary Fig. S2). We also confirmed that *tisB* expression levels were comparable between the different constructs and that no major mRNA degradation occurred (Supplementary Fig. S3). Since the 3xFLAG-tag attenuates TisB functionality (data not shown), we used the p0SD-*tisB* plasmid for all subsequent physiological experiments.

It is known that cell growth is inhibited upon induction of *tisB* expression from plasmids^15,16,23^. Growth curve analysis demonstrated that wild-type TisB stopped cell growth within 30 min after its induction during exponential phase (Fig. 1d). While the K29L variant still caused full growth inhibition, the D5L, D22L and K26L variants were functionally attenuated, as displayed by an incomplete growth inhibition. In contrast, growth inhibition did not occur with the K12L and Q19L variants; growth was comparable to the empty vector control (Fig. 1d). In order to determine whether growth inhibition was accompanied by ATP depletion, intracellular ATP levels were quantified. A one-hour induction of wild-type TisB with L-ara led to a ~ 30-fold decrease of intracellular ATP levels, whereas the empty vector control was unaffected by the L-ara treatment (Fig. 1e). ATP measurements of strains producing TisB variants largely conformed to the growth inhibition data, with D5L being the only exception. While the D22L and K26L variants showed intermediate ATP depletion of ~ 5-fold, the D5L and K29L variants caused ATP depletion comparable to wild-type TisB. In contrast, the K12L and Q19L variants only caused minor ATP depletion of less than 2-fold (Fig. 1e). From these experiments we conclude that K12 and Q19 are mandatory for full growth inhibition, which is concomitant with substantial ATP depletion.

**Fig. 2:**
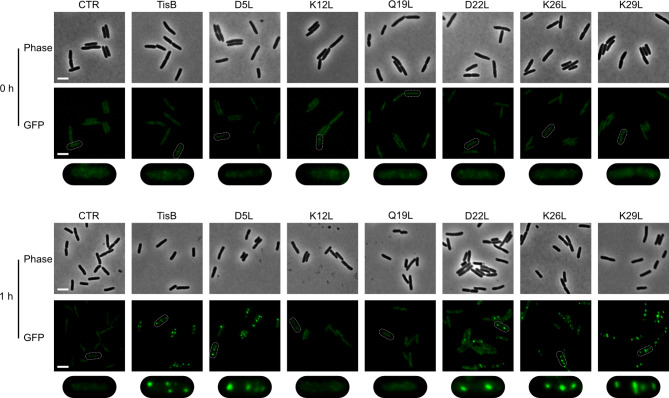
TisB-dependent protein aggregation. Reporter strain MG1655 *ibpA-msfGFP*, harboring p0SD-*tisB* (TisB) and variants with different amino acid substitutions, were treated with L-ara (0.2%) during exponential phase for one hour. An empty pBAD plasmid (CTR) was used as control. Pre- and post-treatment samples were analyzed by microscopy. Phase contrast (phase) images are displayed together with corresponding fluorescence images (GFP). White bars represent a length scale of 2 μm. The area surrounding a single cell observed in the GFP image (white dashed line) is magnified and shown below the original images for closer inspection.

It has been shown that ATP depletion is a major driving force for protein aggregation in bacteria, which in turn impacts the duration of the dormant state^24,25^, and we have recently observed that TisB is able to cause protein aggregation in *E. coli* MG1655^16^. To further validate functionality of the TisB variants, we tested their ability to cause protein aggregation by applying an established reporter strain that produces a monomeric superfolder GFP (msfGFP) fused to the small heat shock protein IbpA^26^. If protein aggregates occur, the IbpA-msfGFP fluorescence changes from a dispersed pattern to the occurrence of distinct cytoplasmic foci. Expression of wild-type *tisB* and its mutant alleles was induced by L-ara for one hour and the formation of IbpA-msfGFP foci was monitored by fluorescence microscopy. Before induction, *E. coli* cells displayed a dispersed fluorescence pattern (Fig. 2). In contrast, induction of wild-type TisB and most TisB variants led to foci formation, which was indicative of protein aggregation. However, foci were absent in the case of K12L and Q19L variants (Fig. 2). These results agree with the growth analysis data and ATP measurements, and further underscore the importance of K12 and Q19 for full TisB functionality.

## TisB-mediated protection against antibiotics is abrogated in lysine 12 and glutamine 19 mutants

**Fig. 3:**
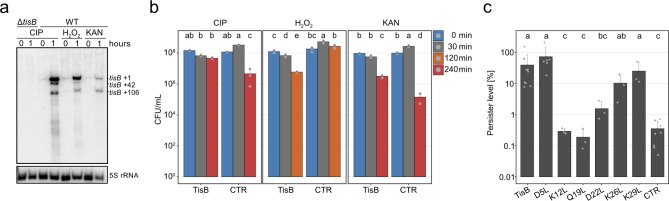
TisB-induced stress tolerance. (**a**) Northern blot analysis of *tisB* induction. Wild type MG1655 (WT) and a *tisB* deletion strain were treated with either 10 µg/mL CIP, 10 mM H_2_O_2_, or 200 µg/mL KAN during exponential phase for one hour. Total RNA was separated using urea-polyacrylamide gels and blotted onto nylon membranes. A radioactive probe was applied for specific *tisB* mRNA detection. Numbers refer to *tisB* primary (+ 1) and processed mRNAs (+ 42 and + 106). 5S rRNA was probed as loading control. For unedited northern blot images, see Supplementary Fig. S3. (**b**) Stress tolerance after TisB induction. Wild type MG1655, harboring either TisB-*tisB* (TisB) or an empty pBAD plasmid (CTR), were treated with L-ara (0.2%) during exponential phase for 30 min to induce *tisB* expression. Cells were subsequently treated with either 10 µg/mL CIP for four hours, 10 mM H_2_O_2_ for two hours, or 200 µg/mL KAN for four hours. Cells before (0 min), after L-ara (30 min), and after treatment with stress agents (120–240 min) were plated on LB agar plates to determine colony counts (CFU/mL). Bars represent the mean of at least two biological replicates (CIP: TisB: *n* = 3; CTR: *n* = 3 | H_2_O_2_: TisB: *n* = 3; CTR: *n* = 3 | KAN: TisB: *n* = 3; CTR: *n* = 2). Error bars indicate the standard deviation. ANOVA with post-hoc Tukey HSD test was performed independently for each treatment, and a compact letter display was applied to present significant groups. (**c**) Persistence induced by TisB. Wild type MG1655, harboring TisB-*tisB* (TisB) and variants with different amino acid substitutions, were treated with L-ara (0.2%) during exponential phase for 30 min to induce *tisB* expression. An empty pBAD plasmid (CTR) was used as control. Cells were subsequently treated with 10 µg/mL CIP for four hours. Cells before L-ara and after CIP were plated on LB agar plates to determine relative persister levels. Bars represent the mean of at least three biological replicates (TisB: *n* = 10; CTR: *n* = 9; D5L: *n* = 6; K12L: *n* = 3; Q19L: *n* = 3; D22L: *n* = 3; K26L: *n* = 3; K29L: *n* = 3). Error bars represent the standard deviation. ANOVA with post-hoc Tukey HSD test was performed, and a compact letter display was applied to present significant groups.

TisB-mediated persistence is a well-established phenomenon, and it has been shown that *tisB* expression increases the number of persister cells after treatment with different antibiotics, such as the gyrase inhibitor ciprofloxacin, the cell wall synthesis inhibitor ampicillin, and the protein synthesis inhibitor streptomycin^18^. Transcription of the native *tisB* gene is strongly induced as part of the SOS response upon treatment with UV light or DNA-damaging drugs, such as ciprofloxacin (CIP)^12–14^. Here, we tested whether transcription of *tisB* is inducible by the ROS hydrogen peroxide (H_2_O_2_) or the protein synthesis inhibitor kanamycin (KAN) in *E. coli* MG1655. H_2_O_2_ was chosen because it is known to induce the SOS response^27^ but has a different mode of action as compared to CIP. KAN was chosen because it is not able to induce the SOS response at sub-inhibitory concentrations in *E. coli*^28^, and we were curious whether a high-dose KAN treatment would lead to induction of *tisB*. Indeed, both H_2_O_2_ and KAN increased the steady-state levels of *tisB* mRNA after one hour of treatment. However, while H_2_O_2_ caused a strong induction that was almost comparable to the CIP treatment, KAN only caused a very slight increase in *tisB* mRNA levels (Fig. 3a). We subsequently tested whether heterologous *tisB* expression from p0SD-*tisB* was able to provide protection against the three different agents. Cultures were first treated with L-ara for 30 min to induce *tisB*, and subsequently treated with one of the agents for 240 min (CIP and KAN) or 120 min (H_2_O_2_). Induction of *tisB* itself led to a slight decrease in colony forming units (CFU), indicating growth stasis and moderate toxicity (Fig. 3b). However, after treatment with the two antibiotics CIP and KAN, an enhanced survival was observed in comparison to the empty vector control (Fig. 3b and Supplementary Fig. S4). Interestingly, TisB did not protect against H_2_O_2_. On the contrary, while the empty vector control was almost unaffected by H_2_O_2_, survival of the *tisB* expression strain showed a 20-fold reduction (Fig. 3b and Supplementary Fig. S4). We conclude that TisB protects against bactericidal antibiotics by promoting a state of cellular inactivity^29^, but that the combination of TisB toxicity and H_2_O_2_ represents a lethal situation, from which it is difficult to recover^15^.

Since wild-type TisB provided almost full protection against CIP (Fig. 3b), we applied this antibiotic to elucidate functionality of the TisB variants. In agreement with the previous experiments, the D5L and K29L variants were fully functional, whereas the K12L and Q19L variants were non-functional. The D22L and K26L variants showed intermediate protection against CIP (Fig. 3c). The results mainly mirrored the ATP measurements (Fig. 1e), suggesting that ATP depletion is one of the main drivers of TisB-mediated inactivity and antibiotic tolerance^30,31^.

## Lysine 12 is essential for TisB functionality and persister formation in the native context

To transfer our observations to the native *tisB* context, we constructed two different *tisB* mutant alleles in the *E. coli* MG1655 chromosome by recombineering techniques: the aspartate at position 5 was replaced with an asparagine (D5N), and the lysine at position 12 was replaced with a leucine (K12L). Based on the previous results, we expected the D5N allele to be fully functional and the K12L allele to be non-functional. Both mutant alleles were compared to wild type MG1655 and a *tisB* deletion strain. To induce *tisB* expression from the chromosomal locus, cultures were subjected to a high-dose CIP treatment (10 µg/mL) for six hours. This condition is known to reveal TisB-dependent effects^16^ and was used here to observe physiological consequences of the chromosomal manipulations.

**Fig. 4:**
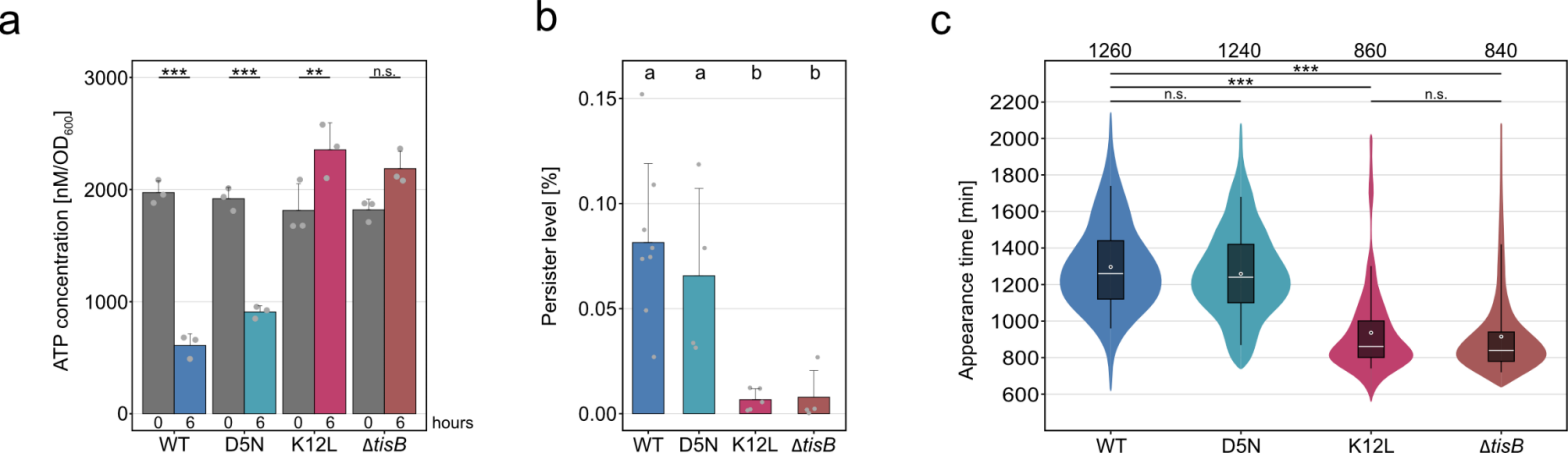
Lysine 12 is essential for TisB-dependent antibiotic persistence. Wild type MG1655 (WT), a *tisB* deletion strain, and two chromosomal amino acid substitutions (D5N and K12L) were treated with CIP (10 µg/mL) during exponential phase for six hours. (**a**) TisB-dependent ATP depletion. Pre- and post-treatment samples were analyzed using a luciferase-based assay to measure cellular ATP levels (nM per OD_600_). Bars represent the mean of three biological replicates. Error bars indicate the standard deviation. ANOVA with post-hoc Tukey HSD test was performed (*** *p* < 0.001; ** *p* < 0.01; n.s.: not significant). (**b**) Persister cell survival. Cells before and after treatment were plated on LB agar plates to determine relative persister levels. Bars represent the mean of at least four biological replicates (WT: *n* = 8; D5N: *n* = 4; K12L: *n* = 5; Δ*tisB*: *n* = 4). Error bars represent the standard deviation. ANOVA with post-hoc Tukey HSD test was performed, and a compact letter display was applied to present significant groups. (**c**) Persister cell recovery. The ScanLag method was applied to determine the colony appearance time after CIP treatment. Colony appearance times are illustrated as violin box plots. Colonies from at least two biological replicates were combined (WT: *n* = 741; D5N: *n* = 930; K12L: *n* = 165; Δ*tisB*: *n* = 375). The white dot indicates the mean. The respective median appearance time (white bar) is shown on top of each plot. Strains were compared using a pairwise Wilcoxon rank sum test (*** *p* < 0.0001; n.s.: not significant).

First, we quantified the ATP levels. In the CIP-treated wild type and D5N strain, ATP levels dropped by 2 to 3-fold, whereas ATP levels even slightly increased in the *tisB* deletion and K12L strain (Fig. 4a). These observations indicate a CIP-induced ATP decline that is based on TisB activity. Next, we determined the persister frequency (Fig. 4b) and the duration of dormancy (Fig. 4c), as measured by the colony appearance time after CIP treatment^32,33^. In both measurements, the D5N strain was comparable to the wild type with a persister frequency of ~ 0.1% and a colony appearance time of ~ 1,250 min. The K12L strain, on the other hand, had a significantly reduced persister frequency of ~ 0.01%, which was comparable to the *tisB* deletion strain (Fig. 4b). Likewise, the colony appearance time was reduced to ~ 850 min (Fig. 4c), demonstrating that functional TisB is needed to increase both the persister frequency and the duration of dormancy. In conclusion, the chromosomal manipulations confirmed our expectations and further strengthened the essentiality of K12.

## A library approach reveals further determinants of TisB functionality

**Fig. 5:**
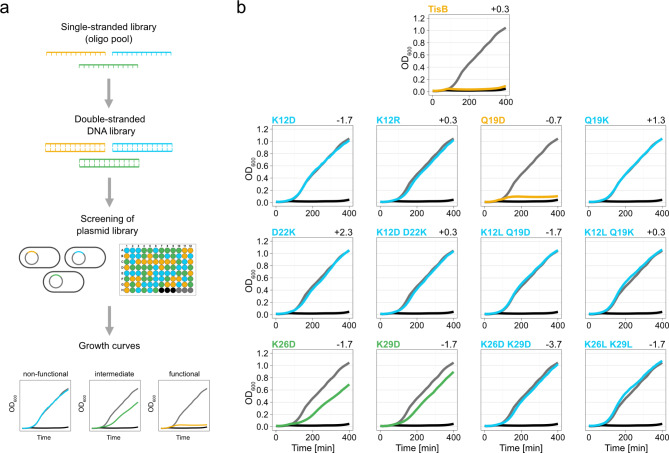
Screening of a *tisB* expression library. (**a**) Schematic representation of generation and screening of the *tisB* expression library. A single-stranded DNA library, representing various *tisB* variants, was converted into a double-stranded DNA library *via* PCR and cloned into expression plasmids. Plasmids were transformed into wild type MG1655 and screened with respect to growth inhibition in the presence of L-ara (0.2%) in a 96-well format. TisB variants were categorized as non-functional (blue), intermediate (green), and functional (yellow). (**b**) Screening of the *tisB* expression library. After growth analysis, results for each TisB variant were combined to generate mean growth curves (native TisB: *n* = 7; K12D: *n* = 2; K12R: *n* = 5; Q19D: *n* = 6; Q19K: *n* = 7; D22K: *n* = 4; K12D D22K: *n* = 2; K12L Q19D: *n* = 7; K12L Q19K: *n* = 5; K26D: *n* = 4; K29D: *n* = 3; K26D K29D: *n* = 4; K26L K29L: *n* = 2). Three biological replicates of p0SD-*tisB* (black) and an empty pBAD plasmid (grey) were included as controls. The individual net charge of each TisB variant (as calculated with the Prot pi Protein Tool) is represented on the top right of each plot.

So far, we were able to demonstrate that replacing some of the charged or polar AAs with the hydrophobic AA leucine led to reduced (D22L and K26L) or abolished TisB functionality (K12L and Q19L). To further reveal the importance of charged AA residues and their positions within the TisB peptide chain, we constructed a small library of expression plasmids containing 12 mutated *tisB* sequences. The wild-type *tisB* sequence was included as a positive control. The single-stranded library (oligo pool) was converted into a double-stranded DNA library and subsequently cloned into plasmid pSL0002 for arabinose-inducible *tisB* expression from the P_BAD_ promoter (Fig. 5a)^34^. The resulting plasmid library was transformed into *E. coli* MG1655, and individual colonies were transferred into a 96-well plate together with empty vector and p0SD-*tisB* controls. The experimental layout was expected to result in a > 6-fold coverage of the library, and sequencing of the plate revealed that each variant was represented by at least two individual clones. After overnight cultivation in regular LB medium, cells were transferred into a new 96-well plate containing LB medium with the inducer L-ara. Growth of individual clones was monitored in a plate reader to assess functionality of the TisB variants (Fig. 5a).

Only the Q19D variant was able to fully inhibit growth, demonstrating that Q19 can be functionally replaced by a negatively charged aspartate. In contrast, replacing Q19 with a positively charged lysine (Q19K) led to a non-functional TisB variant (Fig. 5b). Neither Q19D nor Q19K were able to compensate for the loss of functionality of the K12L substitution. Furthermore, we were not able to obtain functionality when replacing K12 with either an aspartate (K12D) or arginine (K12R), demonstrating that TisB has a strict requirement for a lysine at position 12 (Fig. 5b). In contrast to the D22L substitution, which still showed intermediate functionality (cf. Fig. 1d), a D22K variant was non-functional and was also not able to compensate the K12D substitution (Fig. 5b). Finally, replacing the positively charged residues at the C-terminus by negatively charged aspartates either attenuated (K26D and K29D single substitutions) or completely abolished functionality (K26D K29D double substitution), indicating the importance of positive charges at the C-terminus. In line with this, a K26L K29L double substitution was non-functional as well (Fig. 5b). According to the ‘anion-selective pore’ model, a net charge of + 1 favors anion selectivity and, hence, TisB functionality^19^. However, we were not able to correlate the net charge of the tested TisB variants with their functionality (Supplementary Fig. S5).

## Discussion

Most type I toxins are membrane proteins with a small size and high hydrophobicity, features that have complicated their direct investigation in living cells. To bypass this limitation, and to learn more about the mode of action, type I toxins have been studied in model lipid bilayers (in vitro) or by molecular dynamics simulations (in silico)^10,19–21,35^. These analyses have pushed forward models of how these toxins organize themselves in the inner membrane to fulfill their functions. In this study, we aimed at substantiating the prevailing in vitro and in silico models for TisB by physiological experiments with *E. coli*.

Both in vitro and in silico models predict that charged and polar AAs are important for TisB functionality. By replacing individual AAs with leucine, we demonstrate that K12 and Q19 are most important for a variety of TisB-dependent phenotypes, including growth inhibition, ATP depletion, protein aggregation, and antibiotic tolerance^16,18,23^. Interestingly, moderate *tisB* expression almost fully protects against the DNA-damaging antibiotic CIP, whereas *tisB* expression is a disadvantage when cells are challenged with oxidative stress by H_2_O_2_. We have shown before that TisB itself provokes generation of ROS, such as superoxide, and that a failure in ROS detoxification may delay or even prevent recovery^15^. We conclude that TisB-dependent dormancy is an efficient means to avoid CIP-induced DNA damage^18,36^, but that the combined effect of endogenous ROS production and H_2_O_2_ administration cannot be efficiently tolerated by TisB-producing cells. Alternatively, TisB might compromise the ability to efficiently detoxify H_2_O_2_, but this needs further investigation.

But how can we accommodate our physiological data with the current TisB models? Gurnev and colleagues applied synthetic TisB for conductance measurements in planar membranes consisting of the model lipid diphytanoyl-phosphatidylcholine^19^. These experiments indicated that TisB forms narrow pores, which maintain cooperativity in honeycomb-like clusters. Based on their experiments with K26A and D5A variants, it was further suggested that a positive net charge promotes anionic selectivity of the TisB pore. According to this in vitro model, hydroxyl anions traverse the inner membrane and discharge the PMF by neutralizing protons in the periplasm. However, our experiments do not support anionic selectivity, since the net charge shows no correlation with TisB functionality in living *E. coli* cells. We conclude that selectivity in the complex environment of a natural membrane may be different than in model membranes. Using pH-sensitive fluorescent proteins it has been demonstrated in *E. coli* that the DNA-damaging antibiotics nalidixic acid and ofloxacin cause loss of pH homeostasis and acidification of the cytoplasm^17,37^. In case of ofloxacin, cytoplasmic acidification could be linked to TisB^17^, and we therefore suggest that TisB acts as a protonophore, which enables discharge of the proton gradient and cytoplasmic acidification. Whether the TisB pore has a rather broad specificity and allows the opposing transition of protons and hydroxyl anions remains an unexplored possibility.

Another intriguing question concerns the oligomeric state of TisB within the inner membrane. In silico analyses by molecular dynamics simulations predict that the basic unit of TisB oligomers is an antiparallel dimer that is stabilized *via* a ladder of salt bridges, a so called ‘charge-zipper’^22^. Subsequent refinements of the simulations suggested that TisB assembles into a tetramer that is best described as an antiparallel dimer-of-dimers^21^. Based on proximity estimations, the model makes the prediction that the basic dimer is mainly stabilized by two K12-D22 salt bridges and one Q19-Q19 hydrogen bond. In support of the ‘charge-zipper’ model, we observed here that both K12D and D22K single substitutions abolished TisB functionality, likely because the mandatory salt bridges were not formed. However, TisB still showed intermediate functionality when D22 was replaced with a leucine. Furthermore, we expected that a D22K substitution should be able to compensate for the functionality loss of a K12D substitution due to establishment of alternative D12-K22 salt bridges. However, the K12D D22K double substitution was not functional, which contradicts the ‘charge-zipper’ model. The Q19 residues were assumed to coordinate both the basic dimer and the dimer-of-dimers through a variety of Q19-Q19 and K12-Q19 hydrogen bonds^21^. Interestingly, we observed that a Q19D substitution was still fully functional, suggesting that K12-D19 salt bridges may compensate for the loss of Q19-mediated hydrogen bonds. In summary, our data supports an essential role of the positively charged K12 residue, since it could not be functionally replaced by any of the tested AAs, not even by the positively charged arginine. We further suggest that K12-Q19 interactions may be more important for TisB oligomerization than K12-D22 interactions but cannot exclude alternative explanations for the observed effects. We conclude that further analyses of native TisB are needed to reveal the oligomeric state of TisB and the particular influence of the charged and polar amino acids.

Replacement of K26 or K29 with a negatively charged aspartate impaired TisB functionality. Similarly, in the case of the type I toxin ZorO (29 AAs) in *E. coli* O157:H7 EDL933, replacement of the C-terminal lysine K29 with a negatively charged glutamate prevented growth inhibition and ATP depletion by ZorO^38^. We further show that TisB has a requirement for at least one positively charged AA (either K26 or K29) at its C-terminus, since substitution of both lysines completely abolished functionality. Similar observations were made for the type I toxin AapA1 in *Helicobacter pylori* (30 AAs). In *H. pylori*, the two C-terminal lysines, K29 and K30, could not be truncated without losing toxicity^35^. Similarly, in *Staphylococcus aureus*, the toxic peptide SprG1_31_ (31 AAs) needs two C-terminal lysines, K30 and K31, for full toxicity. Interestingly, the two lysines could not be swapped to the N-terminus to retain toxicity^39^. These observations raise the question of how the C-terminal positive charges, which are not integral to the TMH, contribute to toxicity. One possibility is that the positively charged C-terminus is important to establish an initial attachment of the toxins to the negatively charged inner membrane, which is followed by hydrophobic interactions and membrane integration^40,41^. The positively charged residues may also provide orientation of the toxins within the membrane due to electrostatic repulsion by protons in the periplasm, which is the foundation of the so-called ‘positive-inside rule’^42^. In case of TisB, the positive charges may accommodate the C-terminus in the cytoplasm, while the N-terminus extends into the periplasm, a so-called N_out_-C_in_ orientation^43^. However, a strict N_out_-C_in_ orientation is not consistent with an antiparallel TisB orientation predicted by the ‘charge-zipper’ model. It also raises the questions whether TisB affects its own orientation as soon as the electrostatic repulsion is alleviated by discharge of the PMF and neutralization of the periplasm. Hypothetically, TisB adopts different PMF-sensitive configurations within the inner membrane, similar to what has been described for phage holins^44^.

Finally, the importance of charged AA residues may differ between different type I toxins. In case of the Fst toxin (33 AAs) in *Enterococcus faecalis*, the charged residues at the N-terminus (K2 and D3) seem to be more important for toxicity than the highly charged C-terminus^45^, albeit progressive truncation of the C-terminus reduced toxicity^46^. The IbsC toxin in *E. coli* is comparably short (19 AAs) and only has one charged residue at its N-terminus (R3), which is dispensable for toxicity^47^. It, therefore, remains challenging to define common rules for type I toxin functionality based on charged AAs. We suggest that high-throughput screens of synthetic toxin libraries, including chimeras of natural toxins and engineered toxins, may help to further dissect the importance of certain amino acids and their positioning within the peptide chain.

### Methods

#### Growth conditions

*E. coli* strains (Supplementary Table S1) were grown in Lysogeny Broth (LB) under conditions of 37 °C and a rotational speed of 180 rpm. When temperature-sensitive plasmids were used, the cultivation temperature was adjusted to 30 °C while maintaining the same rotational speed. Antibiotics were added to pre-cultures as required, with concentrations of 50 µg/mL for kanamycin, 15 µg/mL for chloramphenicol, 200 µg/mL for ampicillin, and 6 µg/mL for tetracycline. Pre-cultures were obtained from single colonies, grown overnight, and subsequently diluted at a ratio of 1:100 into fresh LB medium to start experimental cultures. The growth curves were monitored in 30-min intervals at 600 nm using a Fisher Scientific cell density meter (model 40).

## Construction of plasmids

Plasmids p0SD-*tisB* and p0SD-3xFLAG-*tisB*^16^ were used as templates for site-directed mutagenesis PCR to generate amino acid substitutions. Primers are listed in Supplementary Table S3. After PCR, the template DNA was digested using DpnI (Thermo Fisher Scientific). The PCR product was transformed into chemically competent MG1655 cells, and clones were selected on LB agar plates containing ampicillin (200 µg/mL). All plasmids were verified by Sanger sequencing (Microsynth SeqLab) and are listed in Supplementary Table S2.

### Chromosomal manipulations

Lambda red recombineering was applied to generate gene deletions and point mutations in the MG1655 chromosome^48^. For expression of λ red genes, the temperature-sensitive plasmid pSIM5 was used^49^. Gene deletions were constructed by insertion of resistance cassettes *via* homologous recombination according to standard protocols^48^. Point mutations were introduced *via* a scarless, two-step λ red protocol using *sacB* as counter-selection marker^14^. The resulting strains (Supplementary Table S1) were verified by diagnostic PCR and Sanger sequencing (Microsynth SeqLab). Primers for λ red recombineering are listed in Supplementary Table S3.

### Determination of colony counts and persister levels

Exponential-phase cultures (OD_600_ ~ 0.4) were treated with L-ara (0.2%) for 60 min to induce *tisB* expression. In case cultures were subsequently treated with CIP (10 µg/mL), H_2_O_2_ (10 mM), or KAN (200 µg/mL), the L-ara treatment was reduced to 30 min. If cultures were treated with CIP (10 µg/mL) alone, the treatment duration was adjusted to six hours. Samples were collected before and after treatments, washed and serially diluted with 20 mM MgSO_4_, and then plated on LB agar plates. The colonies were counted after approximately 20 h (pre-treatment samples) or 40 h (post-treatment samples). The colony counts were used to calculate the colony forming units per milliliter (CFU/mL). The survival upon CIP treatment (i.e., persister level) was determined by calculating the ratio of treated and untreated samples. *P*-values were calculated using an ANOVA followed by a post-hoc Tukey’s HSD test, implemented in R statistical language (https://www.r-project.org/).

### Analysis of colony growth

The ScanLag method was used to analyze colony growth^32^. LB agar plates from CIP experiments were covered with black felt, positioned on scanners, and incubated at a temperature of 37 °C. Time series of images were captured using Epson Perfection V39 scanners under the control of the *ScanningManager* application^50^. TIFF files were generated every 20 min over a total duration of 40 h. Image processing was performed using MatLab (MathWorks), using functions *PreparePictures*, *setMaskApp*, *TimeLapse*, and *ScanLagApp*. After image processing, the appearance and growth times were extracted. The time of appearance is characterized by a colony size of 10 pixels, while the growth time is defined as the duration required for a colony size to increase from 80 to 160 pixels. The data was used to construct violin box plots *via* Power BI Desktop (Microsoft). *P*-values were calculated using a pairwise Wilcoxon rank sum test in R statistical language (https://www.r-project.org/).

### ATP measurements

Exponential-phase cultures (OD_600_ ~ 0.4) were treated with L-ara (0.2%) for one hour or with CIP (10 µg/µL) for six hours. Pre- and post-treatment samples (1 mL) were extracted. Cell pellets were harvested *via* centrifugation (13,000 rpm, 3 min), and the supernatants were discarded. The cells were rinsed with 1 mL of NaCl (0.9%) and resuspended in 1 mL of LB medium. A mixture was prepared by combining 100 µL of the samples with 100 µL of BacTiter-Glo reagent (Promega). Mixtures were incubated for 5 min in the dark. The luminescence was quantified using an Infinite M Nano + microplate reader (Tecan). The values obtained were converted to nM, using the slope formula of an ATP calibration curve, and normalized to the OD_600_. *P*-values were calculated using an ANOVA followed by a post-hoc Tukey’s HSD test, implemented in R statistical language (https://www.r-project.org/).

### Fluorescence microscopy

Exponential-phase cultures (OD_600_ ~ 0.4) were treated with L-ara (0.2%) for 60 min. Samples were collected both prior to and post-treatment. Samples were then placed on 1% agarose pads in 1x phosphate-buffered saline (PBS). The agarose pads were positioned on a microscopy slide, and the cells were covered with a cover slip. Imaging was performed using a Leica DMI6000 B inverted microscope, equipped with an HCX PL APO 100x/1.4 differential interference contrast (DIC) objective. The images were captured using a pco.edge sCMOS camera and processed with VisiView software (version 4.3.0, Visitron Systems GmbH). For fluorescence images (GFP), a custom filter set was used (T495lpxr, EX470/40m; EM525/50, Chroma Technology). The exposure time was set to 50 ms, with a binning factor of 2 and an offset of 0.0. The captured images were saved in the TIFF format and subsequently processed using the open-source software ImageJ (version 1.53k).

### Northern blot analysis

Exponential-phase cultures (OD_600_ ~ 0.4) were treated with CIP (10 µg/mL), H_2_O_2_ (10 mM), or KAN (200 µg/mL) for one hour. Total RNA of pre- and post-treatment samples was isolated using the acid-phenol method as described^13^. Northern blot analysis was performed with 5 µg of total RNA. The RNA was separated using a 10% polyacrylamide gel, containing 1x TBE and 7 M urea. Separation was done at 300 V for an approximate duration of three hours. The separated RNA was transferred onto a Roti Nylon plus membrane (Roth) *via* semi-dry electroblotting at 250 mA for three hours, followed by UV-crosslinking. The membrane was pre-hybridized using Church buffer [0.5 M phosphate buffer (pH 7.2), 1% (w/v) bovine serum albumin, 1 mM EDTA, 7% (w/v) SDS] at a temperature of 42 °C for one hour. Hybridization with radioactively labelled probes was performed overnight at the same temperature. Specific probes were generated by end-labeling of oligodeoxyribonucleotides (Supplementary Table S3) using T4 Polynucleotide Kinase (New England Biolabs) and [γ-^32^P]-ATP (Hartmann Analytic). After hybridization, membranes were washed (5x SSC, 0.01% SDS) and exposed to phosphorimaging screens (Bio-Rad). Screens were analyzed using a Molecular Imager FX and the Quantity One 1-D Analysis Software (Bio-Rad).

### Sample generation for western blot analysis

For detection of 3xFLAG-TisB, exponential-phase cultures (OD_600_ ~ 0.4) were treated with L-ara (0.2%) for one hour. Pre- and post-treatment samples (40 mL) were collected, pelleted at 10,000 rpm and 4 °C for 10 min, washed with 2 mL NaCl (0,9%), and centrifuged at 13,000 rpm and 4 °C for 3 min. Cells were washed with cold phosphate buffer (50 mM), centrifuged as before, and subsequently resuspended in 4 mL cold phosphate buffer (50 mM). Cells were lysed by sonication (3 × 30 s, 7 cycles, 70%) using a Bandelin Sonopuls and the ultrasonic immersion probe MS 73. Lysates were centrifuged at 600 rpm for 10 min to remove cell debris. Cytoplasmic and membrane fractions were separated using ultracentrifugation of the supernatants at 105,000 x *g* for 45 min at 4 °C. Supernatants were transferred to new tubes, while the pellets were dissolved in 3 mL 50 mM phosphate buffer containing 0.2% sodium lauroyl sarcosinate. Samples were incubated up to 2 h at room temperature to solubilize membrane proteins. Protein concentrations were determined using the Bradford assay. Proteins were precipitated overnight using four times the sample volume of cold acetone. Samples were centrifuged at 13,000 rpm and 4 °C for 10 min and washed two times with 500 µL cold acetone. Afterwards, acetone was removed by allowing evaporation at room temperature for 15 min. Proteins were adjusted to a concentration of 5 µg/µL in phosphate buffer (50 mM). Finally, SDS sample buffer (12% SDS, 6% β-mercaptoethanol, 30% glycerol, 0.05% Coomassie blue, 150 mM Tris/HCl at pH 7.0) was added for a final concentration of 2.5 µg/µL.

### Western blot analysis

Protein separation was performed using Tricine-SDS-PAGE with a 16% separation polyacrylamide gel and 6% collection polyacrylamide gel. Prior loading, samples with SDS buffer were incubated at 37 °C for 15 min (boiling of samples was avoided because it may cause aggregation of membrane proteins). An initial voltage of 100 V was applied until samples entered the separation gel, after which electrophoresis was conducted at 300 V for approximately three hours. Proteins were transferred onto a PVDF membrane *via* semi-dry electroblotting overnight at 0.4 mA/cm^2^. Membranes were subsequently stained with Ponceau S solution and documented. Membranes were blocked with 5% milk powder in 1x PBST (PBS + 0.1% Tween20) for one hour. For detection of 3xFLAG-TisB, membranes were incubated with an HRP-conjugated monoclonal IgG α-FLAG antibody (Sigma-Aldrich) in 1x PBST with 3% BSA at room temperature for 90 min, followed by visualization using the Lumi-Light Western Blotting Substrate (Roche). For detection of YidC and YchF, membranes were incubated with a rabbit α-YidC and a rabbit α-YchF antibody, respectively, in 1x PBST with 3% BSA at room temperature for 90 min. Subsequently, membranes were incubated with an alkaline phosphatase-conjugated goat α-rabbit antibody in 1x PBST with 3% BSA at room temperature for 90 min. For visualization, CDP-Star (Roche) and AP-buffer (0.1 M Tris/HCl, 0.1 M NaCl, pH 9.5) were used. All signals were documented using a chemiluminescence imager (PeqLab) with the FusionCapt Advance software (Vilber Lourmat). For sequential detection of proteins, membranes were stripped using stripping buffer (0.1 M glycine, 0,37% HCl) at room temperature for one hour. After stripping, membranes were blocked once again with 5% milk powder in 1x PBST for 90 min.

### Construction and analysis of a *tisB* expression library

The *tisB* library was ordered as an oligo pool from IDT (Integrated DNA Technologies). All *tisB* sequences contained the alternative 5’ untranslated region (UTR) from plasmid p0SD-*tisB* for moderate *tisB* expression^16^. In addition, each sequence was extended by universal 5’ and 3’ adapter sequences containing BbsI recognition and primer binding sites (Supplementary Table S4). The single-stranded oligo pool was converted into a double-stranded DNA library by PCR using primers targeted towards the adapter sequences (Supplementary Table S3). The DNA library was subsequently cloned into plasmid pSL0002 using Golden Gate cloning as described elsewhere^34^. The resulting plasmid library was transformed into electrocompetent *E. coli* MG1655 cells. Ninety individual colonies were transferred into a transparent 96-well plate containing 150 µL LB medium per well. Strains containing either an empty vector or p0SD-*tisB* were loaded in triplicates and used as controls. The plate was incubated overnight at 37 °C and 180 rpm, and subsequently used for Sanger sequencing (Microsynth SeqLab). In addition, the overnight plate was used to inoculate two new 96-well plates; the first containing LB medium (growth control plate) and the second containing LB medium with L-ara (0.2%) for induction of *tisB* expression. Growth was monitored at 600 nm in an Infinite M Nano + microplate reader (Tecan) adjusted to 37 °C and orbital shaking (amplitude 3.5 mm). Data was evaluated using the *growthcurver* package in R Studio.

## Electronic supplementary material

Below is the link to the electronic supplementary material.

Supplementary Material 1

Supplementary Material 2

## Data Availability

Datasets are available from the corresponding author upon reasonable request.
