# Supplementary material for: Relevance of charged and polar amino acids for functionality of membrane toxin TisB
Source: Sci Rep. 2024 Oct 3;14:22998. doi: 10.1038/s41598-024-73879-7 (PMC11449926; doi:10.1038/s41598-024-73879-7)
Supplement: Supplementary file 2 — Supplementary Material 2 [file 41598_2024_73879_MOESM2_ESM.pdf]

Supplementary Figures

Relevance of charged and polar amino acids for functionality of membrane toxin TisB

Florian H. Leinberger and Bork A. Berghoff

This file contains:

Figures S1 to S5

Figure S1

Name	Sequence	GRAVY	Net Charge
TisB	MNLVDIAILILKLIVAALQLLDAVLKYLK	1.62	+0.3
D5L	MNLVLIAILILKLIVAALQLLDAVLKYLK	1.87	+1.3
K12L	MNLVDIAILILLLIVAALQLLDAVLKYLK	1.88	-0.7
Q19L	MNLVDIAILILKLIVAALLLLDAVLKYLK	1.87	+0.3
D22L	MNLVDIAILILKLIVAALQLLLAVLKYLK	1.87	+1.3
K26L	MNLVDIAILILKLIVAALQLLDAVLLYLK	1.88	-0.7
K29L	MNLVDIAILILKLIVAALQLLDAVLKYLL	1.88	-0.7
K12D	MNLVDIAILILDLIVAALQLLDAVLKYLK	1.63	-1.7
K12R	MNLVDIAILILRLIVAALQLLDAVLKYLK	1.60	+0.3
Q19D	MNLVDIAILILKLIVAALDLLDAVLKYLK	1.62	-0.7
Q19K	MNLVDIAILILKLIVAALKLLDAVLKYLK	1.60	+1.3
D22K	MNLVDIAILILKLIVAALQLLKAVLKYLK	1.60	+2.3
K12D D22K	MNLVDIAILILDLIVAALQLLKAVLKYLK	1.62	+0.3
K12L Q19D	MNLVDIAILILLLIVAALDLLDAVLKYLK	1.88	-1.7
K12L Q19K	MNLVDIAILILLLIVAALKLLDAVLKYLK	1.87	+0.3
K26D	MNLVDIAILILKLIVAALQLLDAVLDYLK	1.63	-1.7
K29D	MNLVDIAILILKLIVAALQLLDAVLKYLD	1.63	-1.7
K26D K29D	MNLVDIAILILKLIVAALQLLDAVLDYLD	1.64	-3.7
K26L K29L	MNLVDIAILILKLIVAALQLLDAVLLYLL	2.15	-1.7

Figure S1. Sequences of TisB variants.

Sequences of the tested TisB variants are shown. Nonpolar amino acids (yellow), polar amino acids (purple), acidic amino acids (red), and basic amino acids (blue). The grand average of hydropathy (GRAVY) value was calculated with the GRAVY CALCULATOR (https://www.gravy-calculator.de/). The net charge was calculated with the Prot pi Protein Tool (https://www.protpi.ch/Calculator/ProteinTool).

Figure S2

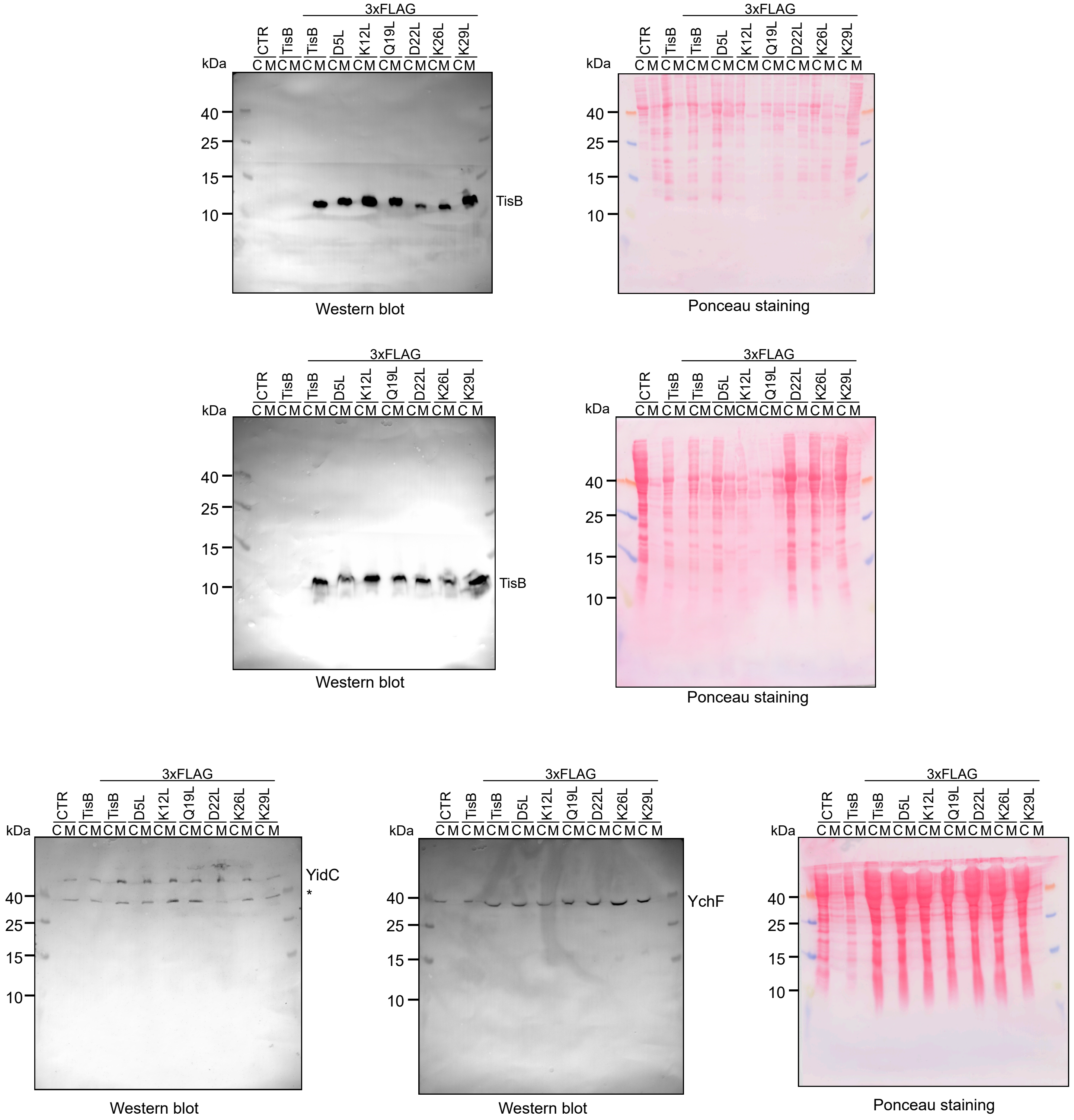

Figure S2. Raw images of western blots.

Unedited western blot and Ponceau staining images shown in Figure 1. Wild type MG1655, harboring p0SD-*3xFLAG-tisB* (3xFLAG-TisB) with different amino acid substitutions, was treated with L-ara (0.2%) during exponential phase. p0SD-*tisB* (TisB) and an empty pBAD plasmid (CTR) were used as controls. Cytoplasmic (C) and membrane (M) fractions were isolated from total protein samples using ultracentrifugation, followed by Tricine-SDS-PAGE. Proteins were blotted onto PVDF membranes. Anti-YidC (membrane) and anti-YchF (cytoplasm) antibodies were used for detection of control proteins. An anti-3xFLAG antibody was used for detection of 3xFLAG-TisB.

Figure S3

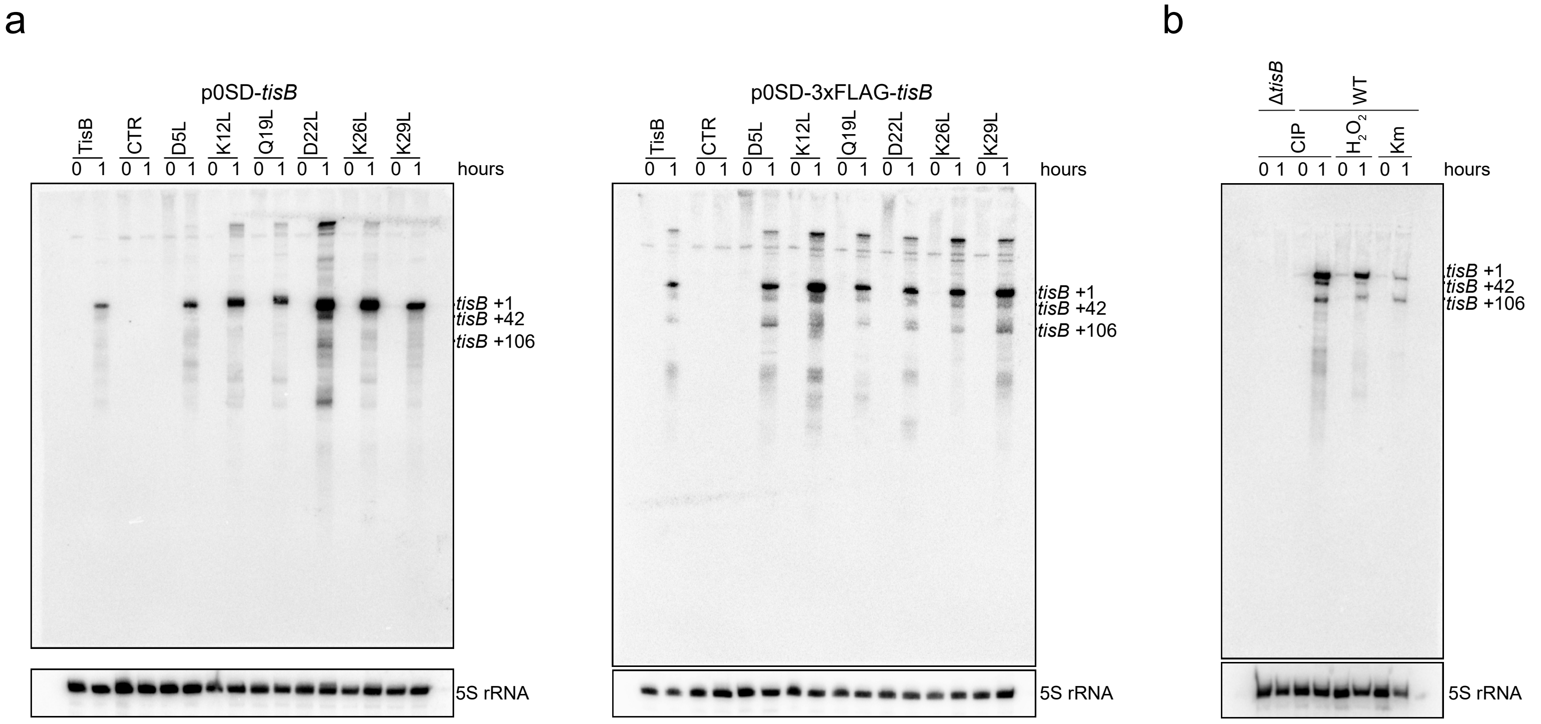

Figure S3. Raw images of northern blots.

(a) Expression analysis of *tisB*. Wild type MG1655, harboring either p0SD-*tisB* or p0SD-*3xFLAG-tisB* with different amino acid substitutions, was treated with L-ara (0.2%) during exponential phase for one hour. An empty pBAD plasmid (CTR) was used as control. Total RNA was separated on urea-polyacrylamide gels and blotted onto nylon membranes. Radioactive probes were applied for detection of *tisB* mRNA and 5S rRNA.

(b) Unedited northern blot images shown in Figure 3a. Wild type MG1655 (WT) and a *tisB* deletion strain were treated with either 10 µg/mL CIP, 10 mM H_2O_2, or 200 µg/mL KAN during exponential phase (OD_{600} ~0.4) for one hour. Total RNA was separated on urea-polyacrylamide gels and blotted onto nylon membranes. Radioactive probes were applied for detection of *tisB* mRNA and 5S rRNA.

Figure S4

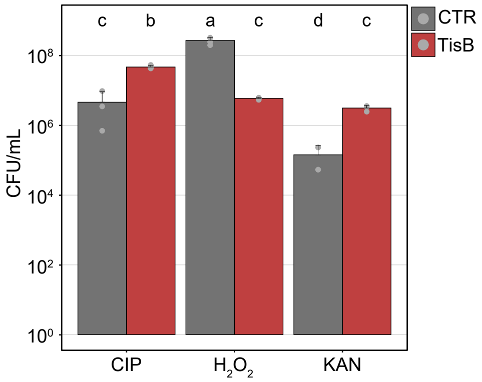

Figure S4. TisB-induced stress tolerance.

Stress tolerance after TisB induction. Wild type MG1655, harboring either p0SD-*tisB* (TisB) or an empty pBAD plasmid (CTR), was treated with L-ara (0.2%) during exponential phase for 30 minutes to induce *tisB* expression. Cells were subsequently treated with either 10 µg/ml CIP for four hours, 10 mM H_2O_2 for two hours, or 200 µg/mL KAN for four hours. After treatment with stress agents, cells were plated on LB agar plates to determine colony counts (CFU/ml). Bars represent the mean of at least two biological replicates (CIP: TisB: n=3; CTR: n=3 | H_2O_2: TisB: n=3; CTR: n=3 | KAN: TisB: n=3; CTR: n=2). Error bars indicate the standard deviation. ANOVA with post-hoc Tukey HSD test was performed, and a compact letter display was applied to present significant groups.

Figure S5

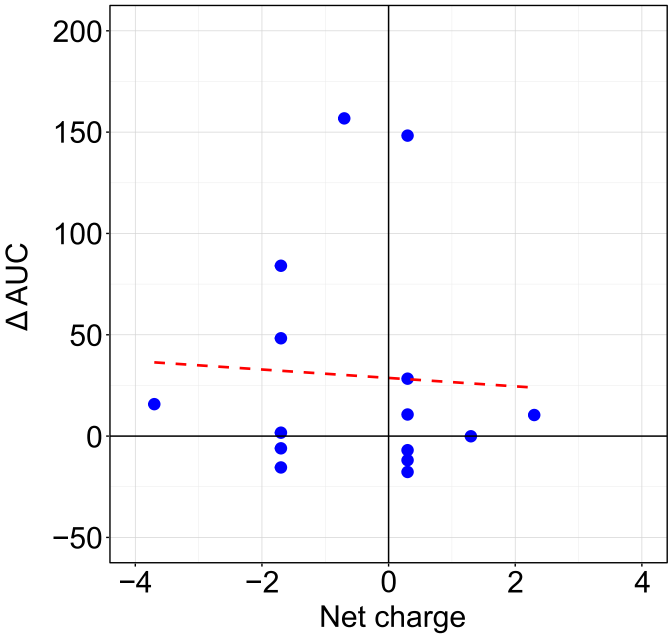

Figure S5. Correlation between net charge and growth inhibition.

Growth curves from Figures 5b were used to determine the area under the curve (AUC) for each TisB variant using the *growthcurver* package in R Studio. An empty pBAD plasmid was used as control to determine ΔAUC values, which were plotted against the net charge of the corresponding TisB variant. The Pearson correlation coefficient (r = -0.056, p = 0.843) was calculated using R statistical language (https://www.r-project.org/).
